# When Should We Think of Myelodysplasia or Bone Marrow Failure in a Thrombocytopenic Patient? A Practical Approach to Diagnosis

**DOI:** 10.3390/jcm10051026

**Published:** 2021-03-02

**Authors:** Nicolas Bonadies, Alicia Rovó, Naomi Porret, Ulrike Bacher

**Affiliations:** 1 Department of Hematology and Central Hematology Laboratory, Inselspital Bern, University of Bern, 3010 Bern, Switzerland; nicolas.bonadies@insel.ch (N.B.); alicia.rovo@insel.ch (A.R.); NaomiAzur.Porret@insel.ch (N.P.); 2 Department for BioMedical Research, University of Bern, 3008 Bern, Switzerland

**Keywords:** thrombocytopenia, myelodysplastic syndromes (MDS), bone marrow failure (BMF) syndromes, aplastic anemia (AA), next-generation sequencing (NGS)

## Abstract

Thrombocytopenia can arise from various conditions, including myelodysplastic syndromes (MDS) and bone marrow failure (BMF) syndromes. Meticulous assessment of the peripheral blood smear, identification of accompanying clinical conditions, and characterization of the clinical course are important for initial assessment of unexplained thrombocytopenia. Increased awareness is required to identify patients with suspected MDS or BMF, who are in need of further investigations by a step-wise approach. Bone marrow cytomorphology, histopathology, and cytogenetics are complemented by myeloid next-generation sequencing (NGS) panels. Such panels are helpful to distinguish reactive cytopenia from clonal conditions. MDS are caused by mutations in the hematopoietic stem/progenitor cells, characterized by cytopenia and dysplasia, and an inherent risk of leukemic progression. Aplastic anemia (AA), the most frequent acquired BMF, is immunologically driven and characterized by an empty bone marrow. Diagnosis remains challenging due to overlaps with other hematological disorders. Congenital BMF, certainly rare in adulthood, can present atypically with thrombocytopenia and can be misdiagnosed. Analyses for chromosome fragility, telomere length, and germline gene sequencing are needed. Interdisciplinary expert teams contribute to diagnosis, prognostication, and choice of therapy for patients with suspected MDS and BMF. With this review we aim to increase the awareness and provide practical approaches for diagnosis of these conditions in suspicious cases presenting with thrombocytopenia.

## 1. Introduction

Thrombocytopenia can be associated with a variety of benign and malignant hematological and non-hematological conditions and the investigation of potential causes is a challenge for clinicians and involved laboratory specialists. Mild and isolated thrombocytopenia (platelet count (PLT) 100–150 G/L) is frequently neglected and not further investigated. In contrast, isolated severe thrombocytopenia (PLT <50 G/L) is often considered as immune-mediated thrombocytopenia (ITP) and treated with steroids without further investigations, in accordance with current guidelines [1]. However, more severe underlying diseases can be potentially missed and the correct diagnosis, consequently, reached with delay. With the currently available diagnostic modalities, the correct and timely identification of myelodysplastic syndromes (MDS) and bone marrow failure (BMF) syndromes can be efficiently done, which is the mainstay for offering patients the most appropriate treatment. With this review we aim to increase the awareness for MDS and BMF, providing practical approaches in suspicious cases presenting with thrombocytopenia.

## 2. Clinical Presentations and Symptoms of MDS and BMF

General practitioners are commonly involved in the initial diagnostic assessment of patients with unclear thrombocytopenia. Thus, their role is crucial in identifying suspicious cases of both MDS and BMF and initiate the correct diagnostic assessments timely. Knowledge on the characteristic clinical features for MDS or BMF are therefore helpful. 

### 2.1. Myelodysplastic Syndromes

With ageing of the general population, and the introduction of next-generation sequencing (NGS) into clinical practice, patients with clonal hematological conditions are increasingly identified. Therefore, in elderly patients with unexplained cytopenia, including isolated thrombocytopenia, clonal disorders, such as MDS and other related myeloid neoplasms, should always be considered as potential differential diagnosis. 

#### 2.1.1. Definition and Pathogenesis of MDS

Patients with unexplained chronic thrombocytopenia should direct awareness towards a potentially underlying myeloid neoplasm, especially in elderly patients with worsening bi- or pancytopenia, or in individuals with previous exposure to chemo- or radiotherapy [2]. MDS are heterogeneous hematopoietic stem and progenitor cell (HSPC) disorders characterized by cytopenia, dysplasia, inflammation, and a propensity to evolve towards secondary acute myeloid leukemia (AML) [3,4]. MDS originate from HSPCs affected by somatic mutations in leukemia-associated genes (SM-LAGs). These mutated HSPC are selected through a stochastic drift that is influenced by a variety of cell-intrinsic and cell-extrinsic mechanisms over a variable duration of time [5]. 

#### 2.1.2. Epidemiology of MDS and Risk Factors

As in many other cancers, MDS and related myeloid disorders are diseases of elderly patients with a median age at presentation above 70 years and male predominance. The exposure to mutagenic agents, such as chemotherapy, radiotherapy, pesticides, insecticides, benzoyl, and solvents, are recognized risk factors [6,7,8,9]. The age-adjusted incidence rate of MDS is between 3–4 per 100,000 patient-years in western countries, with an increase of the age-specific incidence rate to more than ten-fold for individuals >70 years of age [2,10,11]. Incident cases of MDS are assumed to rise substantially in the forthcoming years, due to population ageing, increased cancer survivorship, and improvements in diagnostic accuracy for the detection of clonal hematopoiesis. MDS can also occur in children and younger to middle-aged adults (aged <50 years), in whom an underlying germline predisposition has to be actively explored [12]. 

#### 2.1.3. Presentation and Symptoms of Patients with MDS

Depending on the cell lineages affected by cytopenia, MDS patients suffer from a variety of symptoms at presentation, which comprise fatigue, dyspnea, tachycardia, bacterial infections, or mucocutaneous bleeding [3]. A substantial number of patients with chronic and mild cytopenia can be asymptomatic. In MDS patients, anemia (usually macrocytic) is the predominant abnormality in the peripheral blood (80–85%), followed by thrombocytopenia (30–65%) and neutropenia (40–50%) [13]. Sometimes, patients present with overlapping features of myelodysplasia with cytopenia as well as myeloproliferation. In such cases, patients can present with splenomegaly and accompanying monocytosis (≥1.0 G/L and ≥10% of all leukocytes) in chronic myelomonocytic leukemia (CMML), granulocytosis, or even thrombocytosis in other rare MDS/MPN (myelodysplastic syndrome/myeloproliferative neoplasm) overlap conditions [14]. The association of clonal hematopoiesis with a broad range of autoinflammatory and autoimmune manifestations is generally underestimated. These manifestations may be of paraneoplastic origin and should direct further investigations for an underlying myeloid or lymphatic malignancy [15,16].

### 2.2. Bone Marrow Failure Syndromes

BMF can be separated in acquired and inherited BMF (iBMF) forms. While acquired forms can affect children and adults, congenital forms are particularly more frequent in children less than five years of age, but also in adolescents and young adults. Unusual presentation of congenital forms may show a late presentation even after the fourth decade of life, may be oligosymptomatic, and can be therefore particularly difficult to diagnose.

#### 2.2.1. Definition and Pathogenesis of BMF

Aplastic anemia (AA) is a rare BMF. This non-malignant disease is characterized by pancytopenia and bone marrow hypoplasia of varying severity. Aplastic anemia will be considered idiopathic when no underlying cause can be identified. Idiopathic AA results from autoimmune mediated destruction of early precursors of hematopoietic cells [17,18]. AA can be related to paroxysmal nocturnal hemoglobinuria (PNH), which is a clonal hematopoietic stem cell disorder with various features including hemolytic anemia, bone marrow failure, and thrombosis. Other pathophysiologic mechanisms may also be involved in secondary forms of AA. Direct damage of hematopoietic cells may occur in patients exposed to irradiation, drugs or chemicals, and may represent an underlying cause of AA. Different viral infections including cytomegalovirus (CMV), Epstein–Barr virus (EBV), dengue virus, parvovirus b19, human herpes virus 6 (HHV6), human immunodeficiency virus (HIV), and disseminated adenovirus infections [19] can be associated with the development of AA. Seronegative hepatitis has been reported in 5–10% of patients with AA [20,21,22]. Accelerated attrition of telomeres [23,24,25] and acquired somatic mutations of genes related to myeloid diseases [26,27,28] are associated with an increased risk of MDS, AML, and early death.

iBMF represent a relevant cause of AA in children and are frequently related to germline mutations (Supplemental Table S1). Evidence of such syndromes may be obvious in patients with BMF. On the other hand, the clinical changes can be subtle and the syndrome can only be diagnosed when the hematological picture becomes manifest with severe cytopenias. In this regard, various syndromes exist. Fanconi anemia (FA) [29] is caused by a defect in the DNA repair mechanisms, predisposing to various tumors. Telomeropathies involve a wide variation of genetic disorders caused by mutations in genes of the telomerase or the DNA damage response complexes. The most characteristic syndrome is dyskeratosis congenita (DC) [25]. Shwachman–Diamond syndrome (SDS) [30] is caused by variants of pathogenic genes that affect the biogenesis and mitosis of ribosomes. Congenital amegakaryocytic thrombocytopenia (CAMT) [31] results mainly from mutations in the oncogene of the virus myeloproliferative leukemia (MPL) responsible for encoding the thrombopoietin receptor. 

#### 2.2.2. Epidemiology of BMF and Risk Factors

AA has a variable geographic distribution, the global incidence ranging from 0.7 to 7.4 cases per million inhabitants per year, with 2- to 3-fold higher rates in Asia than in Europe and the U.S. The disease can present at any age, but the presentation is bimodal with a peak in young adults and the elderly. AA affects both sexes in similar proportions [32,33]. Ethnicity and some specific human leukocyte antigen (HLA) characteristics have been associated with higher predisposition to develop AA, higher frequency of small PNH clones and response to immunosuppression [34,35,36,37,38]. Environmental factors may be relevant, such as frequent exposure to benzene-based products [39]. Clonal hematopoiesis is prevalent in AA, and some mutations impact on clinical outcomes. At present, however, prediction of clinical significance is often difficult [40]. The iBMF appear mainly between 2 and 5 years of life, but can manifest at any age during childhood or adolescence. More rarely, diagnosis will be reached later in life [41]. 

#### 2.2.3. General Presentation and Symptoms of Patients with BMF

Patients with AA usually refer symptoms of onset in the previous weeks that are related to the type and number of cytopenias. Symptoms are frequently related to anemia, mainly fatigue, and dyspnea on exertion. Bleeding symptoms are also common including easy bruising, menorrhagia, skin or enoral petechiae typically occurring when thrombocytopenia is significant. Although less frequent, infections can be life-threatening, mainly affecting patients with profound neutropenia [18,42]. Other than that, the clinical examination is classically negative, with absence of lymphadenopathy, splenomegaly or hepatomegaly.

In AA patients, a positive family history including other members affected with cytopenia or malignancy suggests an iBMF. The presence of abnormal clinical features, such as short stature, thumb, or radial ray and/or skeletal abnormalities, microcephaly, hypo- or hyperpigmentation (café au lait spots), eye, renal, or gonadal malformations are changes related to FA [29]. The presence of abnormal pigmentation of the skin, nail dystrophy affecting hands and feet, and oral leukoplakia is considered to be the classic presentation of DC. In addition, premature gray hair and pulmonary fibrosis have been reported [43]. SDS can have a broad clinical phenotype with skeletal abnormalities, steatorrhea consequently to exocrine pancreatic dysfunction, and recurrent infections [30]. CAMT presents with isolated thrombocytopenia and reduced megakaryocytes in the bone marrow without birth defects characteristic of other iBMF [31].

## 3. Diagnostic Approach for MDS and BMF

**Figure 1:**
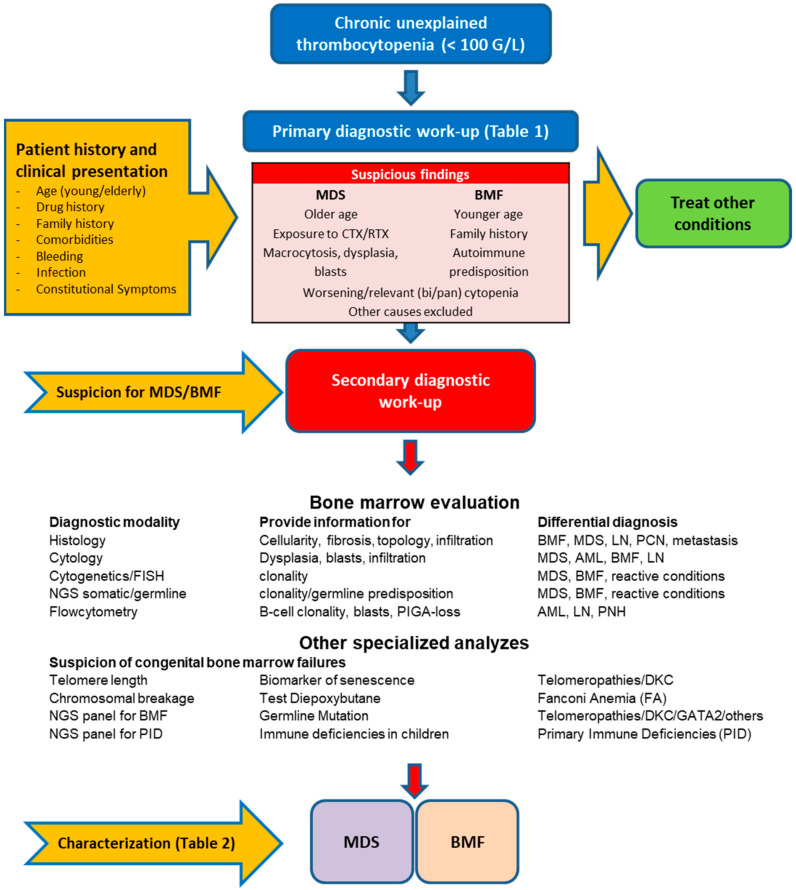
Stepwise diagnostic approach to thrombocytopenia in patients with suspected myelodysplastic syndromes (MDS) or bone marrow failure (BMF) syndromes.

In the following, we will describe our suggest stepwise diagnostic approach, as summarized in Figure 1.

### 3.1. Primary Diagnostic Work-Up

The exclusion of other conditions associated with thrombocytopenia is the mainstay for the primary work up (Table 1). Complete blood counts (CBC) including white blood count (WBC) with a full differential, red blood cell (RBC) analysis of hemoglobin, hematocrit, and RBC indices (including mean cellular volume, MCV) and reticulocyte counts are essential. In patients with MDS or BMF, peripheral blood parameters reveal evidence of different combinations of cytopenia. Anemia is frequently macrocytic (increased MCV), and reticulocytes are usually significantly reduced [44]. The immature platelet fraction (IPF) can be helpful to identify younger platelets and is increased in peripheral consumption. Microscopic cytomorphologic examination of the peripheral blood smear has to be done in cases with unexplained cytopenia to identify morphological RBC abnormalities (schistocytes, anisocytosis, and poikilocytosis), dysplasia, or the presence of cell line precursors and blasts.

Substrate deficiency should be excluded by determination of serum folate, transcobalamin, iron, total iron binding capacity (TIBC), and ferritin. Parameters that may be indicative for hemolysis, such an increased rate of reticulocytes, lactate dehydrogenase (LDH), and bilirubin combined with decreased haptoglobin, with or without a positive direct antiglobulin test (DAT/direct Coombs test), should cast suspicion on hemolytic anemia (PNH, autoimmune hemolytic anemia). Viral infections such as HIV, Parvovirus B19, CMV, EBV, hepatitis B (HBV), and C virus (HCV) should be excluded. Lymphoid neoplasms or plasma cell neoplasms can be identified with protein electrophoresis, immunofixation and free light chain assays. Thyroid-stimulating hormone (TSH), antinuclear antibodies (ANA), antineutrophil cytoplasmic antibodies (ANCA) and rheumatoid factor (RF) direct towards a potential rheumatological disorder and abdominal ultrasound might identify an underlying liver disease, splenomegaly, or lymphoma. Thalassemia and other forms of hereditary hemoglobinopathies should be excluded according to ethnicity as well as personal and family history. 

### 3.2. Secondary Diagnostic Work-Up

Patients with suspicious findings with worsening/relevant cytopenia and exclusion of other causes in the primary work-up should be referred to specialized centers for more detailed investigations. Morphological evaluation of peripheral blood (PB), bone marrow (BM) histology, and cytology with iron staining/assessment as well as cytogenetic analysis are mandatory for the assessment of MDS and BMF. Cytogenetics is indispensable to determine clonality and assess the disease-based risk in case of MDS [45,46]. Currently, asservation of bone-marrow samples for eventual molecular diagnostics with NGS is also advisable. NGS with myeloid panels is instrumental in all patients with unclear cytopenia as well as MDS with normal cytogenetics since it might prove clonality, refine prognosis, contribute to predicting treatment response, and serve as measurable residual disease (MRD) marker after allogeneic hematopoietic stem cell transplantation (allo HSCT) [47]. 

### 3.3. Peripheral Blood Smear

Bi- or pancytopenia is common in both MDS and BMF; however, at their onset, single cell lineages can also be affected. Non-regenerative anemia is almost a constant finding, with suppression of reticulocyte production. RBC macrocytosis is a common feature for MDS and BMF; however, relevant anisocytosis and/or poikilocytosis is predominantly found in MDS [42]. The observation of iron deficiency in the presence of pancytopenia should direct the investigations towards PNH. A normal WBC does not exclude an abnormal differentiation with neutropenia. Neutropenia can occur in varying degrees of severity. Lymphocyte count is generally decreased in MDS but normal in BMF [48]. In some cases, an expansion of large granular lymphocyte (LGL) can accompany AA. Its clinical relevance remains frequently unclear and it can be difficult to distinguish from LGL leukemia [41,49,50]. Monocytopenia may expand the differential diagnosis to hairy cell leukemia. Cytomorphologic examination of the peripheral blood smear has to be done microscopically to assess for morphologic abnormalities of RBC, dysgranulopoiesis and abnormal platelets. The presence of hematopoietic precursors with or without blasts is suggestive for an underling chronic myeloid neoplasm, whereas blasts without hematopoietic precursors (hiatus leucaemicus) is characteristic of acute leukemia. Atypical lymphocytes, e.g., hairy cells, suggest a lymphoproliferative disorder [42,51].

### 3.4. Bone Marrow Cytomorphology 

Bone marrow cyto- (aspirate) and histomorphology (biopsy) are essential and complementary for the diagnosis of MDS and BMF. The examination of bone marrow smears reveals quantitative information about cellularity, assessment of the different hematopoietic lineages (granulopoiesis, erythropoiesis, megakaryopoiesis), and the maturation stages. Increase of blasts or infiltrations of pathologic cell populations may be identified. Scant amount of bone marrow particles can be found in BMF and sometimes in MDS; however, a dry tap is unusual in BMF and suggests other diagnoses [52]. Quantitative and qualitative dysplastic morphological alterations of bone marrow precursors and peripheral blood cells are still fundamental for classification of MDS [44]. According to the 2016 World Health Organization (WHO) update, the presence of at least 10% of dysplastic cells in at least one hematopoietic lineage in the bone marrow is sufficient for a diagnosis of MDS [53]. Nevertheless, dysplastic features of hematopoiesis occur also in healthy individuals. On the other hand, substantial clonal hematopoiesis may exist in cases with less than 10% dysplasia. Following iron staining of the bone marrow smears, evidence of more than 15% ring sideroblasts (that result from mitochondrial iron accumulation) [44] or, in the presence of characteristic *SF3B1* mutations, more than 5%, is a diagnostic criterion for MDS with ring sideroblasts). Increase of marrow myeloblasts to 5% to 19% assign cases to advanced MDS with excess of blasts 1 and 2 (EB-1 or EB-2).

### 3.5. Bone Marrow Histopathology 

Bone marrow trephine biopsies reveal information on cellularity, lineage distribution within the marrow space, and stroma fibrosis. In addition, bone marrow biopsy improves the characterization of megakaryocytes, blast quantification, and characterization of clusters of blasts: this phenomenon is known as “atypical localization of immature precursors” (ALIP). Identification of MDS with fibrosis and also hypoplastic MDS (hMDS) is rendered possible [44]. Likewise, histopathology is essential for the discrimination of MDS cases from overlapping disorders. For better discrimination of MDS from CMML, monocytic cells can be identified by immunohistochemistry, e.g., staining for CD68. Histopathology is crucial for the diagnosis of BMF and requires representative and sometimes repetitive sampling [54]. Aplastic and hypocellular BM is defined by a cellularity below 10% or 30%, respectively. The quality of the trephine biopsy is particularly important in the elderly, who have physiologically hypocellular marrow [55]. A typical AA marrow presents with variable amounts of residual hematopoietic cells with large fat spaces. Abundant plasma cells, lymphocyte and mast cell hyperplasia accompany the picture. Stromal cell hyperplasia can simulate normal cellularity and the increase in lymphocytes and/or mast cells sometimes pretend an infiltrative character. In such cases, immunohistochemistry or flow cytometry may be required to rule out a lymphoid neoplasms or mastocytosis [56]. Erythropoiesis nests forming “hot spots” are characteristic as well [51]. A certain degree of dyserythropoiesis with megaloblastic changes is frequently found in AA and needs to be carefully distinguished from MDS. Granulopoiesis and megakaryocytes are usually severely diminished or absent, without relevant dysplastic changes. Immunohistological staining allows the identification, quantification as well as topographic distribution of blasts, megakaryocytes, abnormal cells and infiltrates. Not infrequently, AA can be associated with lymphoproliferative disorders [56,57,58].

### 3.6. Multiparameter Flow Cytometry 

For MDS, flow cytometric scores have been developed to contribute to the diagnostic process [59]. Dysplastic changes can be identified, e.g., by sophisticated interpretation of surface marker abnormalities in the myeloid compartment, and immature progenitor compartments can be identified. However, NGS seems to replace flow cytometry increasingly for the detection of clonality in MDS [60]. Nonetheless, flow cytometry remains essential to exclude other diagnoses, such as hairy cell leukemia. In patients with AA or hypoplastic MDS, subclones of PNH may be identified, which contribute in confirming the diagnosis. PNH clones are characterized by absence or severe deficiency of glycosylphosphatidylinositol (GPI)-anchored proteins, CD55, and CD59. Loss of the respective antigens is detected by staining with monoclonal antibodies and a reagent known as fluorescent aerolysin (FLAER) [61]. 

### 3.7. Cytogenetics

In around 50% of patients with de novo MDS and in around 80% of patients with therapy-associated MDS (t-MDS), clonal cytogenetic aberrations can be identified by chromosome banding analyses. Entities, such as MDS with isolated 5q deletion according to the WHO classification, can only be defined by karyotyping. Other examples of typical clonal cytogenetic alterations in MDS are abnormalities of chromosomes 7 or 17p. It should be considered that loss of the Y chromosome can be either clonal or age-related in male patients, depending on the proportion of aberrant metaphases [53]. Additionally, the karyotype has a central role for the revised International Prognostic Scoring System (IPSS-R) that discriminates five cytogenetic risk groups [46,62]. In AA, cytogenetic abnormalities can be present in up to 15% of patients [63]. Frequent anomalies include trisomy 8, uniparental disomy of 6p, 5q-, anomalies of chromosome 7 and 13. While abnormalities of chromosome 5 and 7 are very consistent with an underlying MDS, the finding of other abnormalities is not diagnostic. AA patients with del(13q) were reported with favorable response to immunosuppression [64,65]. Fluorescence in situ hybridization (FISH) allows detecting chromosome abnormalities of interphase nuclei also in the case of insufficient chromosome banding analysis. Vital cells are not required for interphase FISH. For patients with suspected or proven MDS, comprehensive interphase probe panels detecting frequent cytogenetic alterations, e.g., of chromosomes 7/7q, 5q, or 17p, may be used. Besides the detection of relevant cytogenetic alterations, FISH allows to confirm or further clarify doubtful results of chromosome banding analysis. At follow-up, the percentage of aberrant interphase nuclei in the case of a previously detected abnormality can be monitored at a sensitive level. Single nucleotide polymorphism (SNP) array analysis allows capturing both DNA copy number and SNP based genotype at a submegabase resolution. This facilitates the detection of small areas of loss of heterozygosity (LOH) of uniparental disomy (UPD) [66] and submicroscopic changes on a cryptic level [60,67]. However, array analyses are increasingly loosing relevance since the introduction of NGS for diagnosis of MDS. 

### 3.8. Next-Generation Sequencing

The detection of SM-LAGs by NGS has gained increasing importance in hematological molecular diagnostics laboratories. NGS allows high-throughput screening for variants that are relevant for diagnosis, classification, risk stratification and, treatment monitoring in patients with hematologic malignancies [68]. At present, targeted sequencing using specific panels covering hotspots of a selection of relevant genes is the method of choice for hematologic diagnostics [69,70]. Examples for commercially available myeloid NGS panels include the Illumina TruSight Myeloid panel (Illumina Switzerland GmBH, Zürich, Switzerland), the Oncomine Myeloid panel (Thermo Fisher Scientific, Reinach, Switzerland), and the Human Myeloid Neoplasms QiaSeq DNA Panel (Qiagen, Rotkreuz, Switzerland). These panels cover hotspots in 25 to more than 50 genes. NGS-targeted sequencing shows a sensitivity between 1% and 5–10%, depending on allele coverage and type of NGS. Each NGS platform has its own technical limitation calling for caution to avoid false positive and negative results. Interpretation of genetic variants relies on thorough assessment using appropriate databases, and the differentiation of a MDS associated mutation from a germline variant needs to be addressed whenever a variant is close to 50% variant allele frequency (VAF). The knowledge on MDS-related markers and the information collected in variant databases are undergoing constant changes, so that variant interpretation can change over time. 

### 3.9. Role of Next-Generation Sequencing in BMF

BMF have a high complexity on the molecular level. The molecular profile may show overlaps with myeloid disorders, rendering the discrimination from MDS difficult. In children and young adults with BMF, it will be necessary to rule out congenital forms by molecular methods, while in older adults, the focus will be more likely on somatic mutations. NGS allows investigating both relevant germline and somatic mutations. The choice of the most appropriate NGS gene panel is of utmost importance. Users of NGS technology should be aware that new pathogenic relevant genes or non-coding regions, like promoter or intronic regions, can be discovered after the design of the gene panel. For this reason, whole exome sequencing is a method of choice for germline panels, allowing a re-analysis of further genes without repetition of the analysis (caveat: non-coding regions are usually missing). Copy number variation (CNV) is a phenomenon frequently observed in BMF where large sections of DNA can be deleted or duplicated; sometimes whole genes are missing. CNV can be detected by array-based comparative genomic hybridization (Array-CGH) technology, which covers large parts of the genome, or multiplex ligation-dependent probe amplification (MLPA) analysis, which focuses on specific genes or genomic regions. Cytogenetic analysis is applied for detection of large chromosomal rearrangements, either by karyotyping (for large rearrangements) or by FISH (for specific rearrangements).

The molecular diagnosis of some iBMF can be straightforward, while others are very complex and heterogeneous [43,71,72,73,74]. DC is heterogeneous and shows complex clinical criteria in concordance with complex genetic findings [75]. For suspected iBMF without a specific clinical pattern, more and more heterogeneous clinical and molecular findings are identified, resulting in newly recognized disease entities [76]. In addition to germline variants, patients with different BMF entities may carry somatic mutations.

### 3.10. Discrimination of Germline from Somatic Mutations 

Somatic NGS analysis in MDS yields a variety of different genetic variants in numerous genes. Most of these variants can be assigned to two groups: first, clonal alterations in relation to the hematologic disorder, and, second, benign germline variants. The first group comprises driver mutations, contributing to the malignant development, and additional passenger mutations. By now, numerous driver mutations are known, including RNA splicing, DNA methylation, transcription, chromatin modification, signal transduction, DNA repair, cohesin complex and associated proteins, RAS pathway, a variety of other signaling molecules, and pathways such as *TP53* [77]. The second group, the benign variants, show always either 50 or 100% VAF, as they are germline variants, unless a somatic deletion at this specific locus has happened, and they are usually listed in databases (UCSC, gnomAD, others). However, there can be variants with a VAF close to 50%, or sometimes distinctly above 50%, which are neither clearly benign nor clonal. Loss of heterozygosity (LOH) needs to be considered in such cases (variant clearly over 50% VAF). Alterations in the *DX41, RUNX1, GATA2*, and *TP53* genes are potentially present in the germline and can cause a predisposition to AML or MDS. For solid tumors, the American College of Medical Genetics (ACMG) developed recommendations for the reporting of presumed germline pathogenic variants (PGPVs) [78]. Confirmatory germline testing should be performed in a specialized laboratory, and positive results have to be explained to the patient by clinicians with genetic expertise. 

## 4. Characterization of MDS and BMF

### 4.1. Challenges in Finding the Diagnosis of MDS and BMF

The approach to suspected MDS of BMF is work-intensive and requires expertise in the interpretation and integration of the laboratory results from various diagnostic modalities, which is best achieved within an interdisciplinary pathological review board.

The conditio sine qua non for MDS is the presence of unexplained cytopenia accompanied by signs of dysplasia in the peripheral blood or bone marrow and, at later stages, increase of immature myeloid blasts. In many instances, cytomorphology alone is not sufficient to confirm or exclude MDS. In such cases, bone marrow cytogenetic analyses can help to identify clonality with chromosome abnormalities in ~50% of all affected patients. NGS has increased the diagnostic sensitivity for the identification of clonal markers of hematopoietic cells in most MDS patients. However, SM-LAGs may also be present in AA, showing some overlap between AA and hMDS, and the distinction may be challenging (Table 2).

AA has phenotypic overlaps with many other hematological disorders, including hypoplastic forms of MDS, AML, and lymphoblastic leukemia (ALL). Moreover, LGL, PNH, and iBMF can manifest with a hypocellular BM. The diagnostic discrimination of these entities is demanding, as AA can occur at any age, lacks specific diagnostic markers, and remains a diagnosis of exclusion. For iBMF, such as telomeropathies and FA, assessment of telomere lengths and chromosome fragility, respectively, are required. (Figure 1). An integrated cyto-histologic/genetic score (hg-score) has been shown to be useful to distinguish hMDS from AA [26,79] (Table 3). The correct diagnosis is especially challenging in asymptomatic patients with moderate thrombocytopenia (PLT 50–100 G/L) and otherwise unsuspicious peripheral blood values. The distinction from immunological, infectious and toxic-reactive causes is critical, as the prognosis and evolution can differ substantially depending on the underlying cause. 

Etiologies can also be multifactoral in elderly patients, i.e., transient aggravation of thrombocytopenia can be observed during infections or drug-exposure in patients with chronic, border-line thrombocytopenia. Delay in recovery after these intercurrences may suggest deficiencies at the HSPC level. 

### 4.2. Characterization of MDS

The challenge to distinguish reactive conditions from early stages of MDS has led an international working group of MDS experts to define minimal diagnostic criteria required for diagnosis of MDS (Table 4) [80]. MDS can develop either primarily or secondarily, after previous radio- or chemotherapy, and are sub-classified according to the 2016 WHO update (Table 5) [53]. Correct MDS sub-classification should be followed by appropriate disease-based and patient-based risk stratification. The International Prognostic Scoring System (IPSS), the revised IPSS (IPSS-R) as well as the WHO Prognostic Scoring System (WPSS) can determine the risk for progression to AML and overall survival [45,46,81]. In order to optimize efficacy against tolerability, patient-derived risk stratification is particularly important for elderly and frail MDS patients. Karnofsky and Eastern Cooperative Oncology Group (ECOG) scores allow assessing the performance status but should be complemented by assessment of comorbidity and frailty. The Charlson Comorbidity Index (CCI) was adapted by Sorror [82,83] and validated for MDS-patients that are sufficiently fit for undergoing allo HSCT (hematopoietic stem cell transplantation comorbidity index: HCT-CI) [84]. A simplified scoring system can be used for elderly MDS patients that considers cardiac, pulmonary, renal and hepatic comorbidities as well as prior treatment for solid tumors as most relevant factors (myelodysplastic syndromes comorbidity index: MDS-CI) [85].

### 4.3. Relevance of Thrombocytopenia in the Context of MDS Patients

Thrombocytopenia in MDS patients is mainly caused by insufficient or ineffective thrombopoiesis, but some patients may have additional immunological mechanisms targeting the mature platelets as well as the megakaryocytic progenitor cells in the bone marrow [86]. In these circumstances, the morphological differential diagnosis between MDS and immune thrombocytopenia (ITP) and the amegakaryocytic form of AA requires identification of characteristic genetic lesions. In retrospective studies, 12% of MDS patients had isolated thrombocytopenia as first presentation [87] and 20% of patients, who were initially classified as ITP, were reclassified with an unusual presentation of MDS [88]. In a review of MDS patients referred to the MD Anderson Cancer Center, 67% of patients were thrombocytopenic (PLT < 100 G/L), of which 26% had moderate (PLT 20–50 G/L) and 17% severe (PLT < 20 G/L) thrombocytopenia [89]. Thrombocytopenia and severe thrombocytopenia were more prominent in higher risk (77% and 20%) compared to lower risk disease (51% and 12%) [89]. The impact of thrombocytopenia on morbidity and mortality is relevant, as half of all MDS patients experience bleeding and a quarter experience even serious bleedings during the course of disease [89,90]. Bleeding episodes can be triggered by treatments with antiplatelet agents or anticoagulants for cardiovascular or thromboembolic comorbidities. Bleeding can be related to quantitative thrombocytopenia (PLT < 10–20 G/L) but also qualitative platelet defects (dysfunctional platelets) [91], caused by somatic or germ-line mutations (i.e., *ETV6*, *RUNX1*, *ANKR1*) [92]. Isolated chronic thrombocytopenia is rare in MDS patients as other lineages are frequently affected either by mild cytopenia or dysplasia [93]. Based on the increased awareness of MDS, earlier hematological assessment of mild cytopenias, and increased use of NGS panels, we and others are currently observing an increasing number of patients with isolated thrombocytopenia as initial manifestation of MDS. Other than that, primary myelofibrosis (PMF) may also present with isolated thrombocytopenia. In such cases, splenomegaly may be detected, accompanied by circulating dakryocytes, myeloid, or erythroid precursors and thrombocyte anisocytosis [53].

### 4.4. Characterization of BMF

The rarity of AA, the difficulties in establishing the correct diagnosis due to the lack of specific markers, and the overlaps with other disorders can delay its diagnosis. When AA occurs in children, congenital forms should be considered. iBMF should be considered in patients with a family history of cytopenias, tendency to cancer, or certain unexplained liver or lung conditions independent of their age. In older adults, the discrimination from hMDS is mandatory and particularly difficult (Table 2 and Table 3).

Identifying the correct underlying disease has implications on the type of treatment. For example, in patients with AA resulting from a nuclear accident as in Chernobyl, allo HSCT is the only option [94], as damage of the hematopoietic cells will not respond to immunosuppressive treatment. Following confirmation of AA and identification of the underlying pathomechanism, its severity must be defined as basis for further therapeutic decisions [95,96].

### 4.5. Isolated Thrombocytopenia as First Presentation of a BMF

Isolated thrombocytopenia will be interpreted and treated as ITP in some patients and only in the course of the disease, a BMF will be finally diagnosed [97]. Nowadays, in accordance with international guidelines, patients with suspected ITP do not necessarily undergo bone marrow investigation [1], and marrow investigations will be done only after failure of standard therapy. Some reports suggested that acquired amegakaryocytic thrombocytopenia may precede AA [98,99,100]. In cases of isolated thrombocytopenia, a detailed personal and family medical history is mandatory, evaluating history of cytopenias, hematological diseases, or a tendency to certain tumors. In a patient with thrombocytopenia, the finding of dysmorphic nails, a history of gray hair early in life, café au lait spots, or any type of physical dysmorphia may suggest an underlying iBMF. Likewise, a history of fibrotic lung disease, liver, or skeletal changes are reasons to consider a consultation in a specialized center [41]. 

### 4.6. Aplastic Anemia and PNH

PNH is a rare bone marrow failure disorder that manifests with hemolytic anemia, thrombosis, and peripheral blood cytopenias. The absence of two glycosylphosphatidylinositol (GPI)-anchored proteins, CD55 and CD59, leads to uncontrolled complement activation that accounts for hemolysis and other PNH manifestations [61]. PNH and AA are related entities [101]. Patients with the typical hemolytic form of PNH can develop AA and around 50% of patients with the acquired form of AA typically have PNH clones [102]. PNH clones accompanying AA are smaller than they are in the PNH hemolytic form. Flow cytometry is the gold standard method for detection and diagnosis of PNH [103]. In AA, PNH clones should be quantified at presentation and at follow-up by serial monitoring every 6 to 12 months, even when the initial result was negative.

### 4.7. Inherited Bone Marrow Failures (iBMF)

In patients with BMF, a positive family history that includes other affected members with cytopenia or malignancy suggests an iBMF. The presence of unusual clinical features (skin, liver, lung, skeletal disease) should alert to the possibility of a congenital form of BMF. A normal clinical examination does not definitively rule out asymptomatic forms of telomeropathies or non-classical presentations of FA. If FA is suspected, investigations that may demonstrate higher sensitivity to chromosomal breakage with mitomycin C or diepoxybutane are necessary [104]. These investigations should be carried out in patients with suspicion of an underlying iBMF. If the mitomycin C or diepoxybutane test is positive, all family candidates to be donors for allo HSCT should also be investigated to rule out asymptomatic forms of FA. In patients with FA, the correct diagnosis is of urgent importance, as less toxic conditioning regimens are mandatory in the case of allo HSCT due to defective DNA repair mechanisms. 

Measurement of the telomere length of peripheral blood leukocytes can be performed as a screening test in case of suspected telomeropathy. A variable percentage of patients without telomeropathies will also show telomere attrition [23,24]. Mutations in the *TERC* and *TERT* genes for example can cause telomeropathies in both children and adults [25]. When telomeropathies occur in adult patients they are more subtle in their clinical presentation, which renders their detection difficult. 

### 4.8. Future Challenges: Unexplained Thrombocytopenia with Clonal Hematopoiesis

After thorough diagnostic assessment, patients with unexplained thrombocytopenia may show insufficient dysplastic morphological changes and lack MDS-defining cytogenetic alterations or sufficient criteria for BMF. These disease forms can be assigned to Idiopathic Cytopenia of Unknown Significance (ICUS), if other clinical conditions are insufficient to explain the cytopenia [80]. In the case that the severity of thrombocytopenia imposes the need of therapeutic intervention, a steroid trial may be justified, whereas in mild to moderate cases observation for 3–6 months is sufficient [105]. As shown in recent years by numerous large studies, SM-LAGs can be identified by NGS in the peripheral blood in an age-dependent, increasing frequency in the elderly population, in up to 20–40% of individuals aged more than 80 years. These mutations are per se not indicative for a hematological neoplasia [106,107,108,109,110], and the affected individuals have generally normal peripheral blood values or only mild cytopenia that do not otherwise fulfill the diagnostic criteria for a hematological malignancy [111]. In case that the variant allele frequency (VAF) of the respective mutations is 2% or more, the condition is termed clonal hematopoiesis with indeterminate potential (CHIP) in otherwise healthy individuals with normal blood values, or clonal cytopenia with unknown significance (CCUS) in individuals with cytopenia, respectively [79,109]. CHIP and CCUS can be considered facultative precanceroses as they are at an increased risk for transformation to overt hematological neoplasms in a rate of 0.5–1% per year [111]. If SM-LAGs present with a VAF level ≥10% or with evidence of two or more somatic associated mutations, a myeloid neoplasm can be diagnosed in patients with unexplained cytopenia with a positive predictive value of >85% [79]. However, with the exception of mutations in spliceosome genes, single mutations in *DNMT3A*, *ASXL1* and *TET2* (DAT mutations) with a VAF <10% are not sufficiently predictive for the diagnosis of a myeloid malignancy, especially in elderly individuals [109]. On the contrary, a negative analysis with a panel of ≥40 genes can exclude a myeloid malignancy with a negative predictive value of >80% [79]. In summary, the advent of NGS has substantially increased the diagnostic sensitivity for detecting clonality and poses some novel challenges in the correct interpretation of these results. Some of those patients will fulfill the criteria for overt myeloid neoplasm as specified above, which will inevitably contribute to steadily increasing incident cases of MDS and associated disorders.

## 5. Conclusions

- Unexplained chronic thrombocytopenia has to be considered as an early and unusual presentation in MDS or BMF.
- Patient’s history remains crucial to identify suspicious cases and for the correct interpretation of primary laboratory values.
- Various diagnostic modalities are required to confirm or exclude MDS or BMF and an interdisciplinary workup is frequently required, especially in difficult cases.
- Meticulous assessment of the PB smear, BM cyto- and histomorphology, as well as cytogenetics are the mainstay of diagnostic evaluation, and is nowadays complemented by NGS and other specialized analyses (telomere length, DNA breakage).
- Repeated bone marrow investigation may be necessary, especially in cases with hypocellular BM for the distinction of sampling errors, reactive-toxic conditions, BMF, and hypoplastic MDS.
- In some occasions, conclusive diagnosis is only possible after follow-up. However, NGS has substantially contributed in identifying early conditions of clonal hematopoiesis, but additional challenges arise for classification and prognostication.

## Figures and Tables

**Table 1:** Primary laboratory evaluation in patients with unexplained thrombocytopenia.

Laboratory Test	Provide Information on
Automated blood count (MPV, IPF), reticulocytes	CBC. Quantitative values. Platelet size and production capacity of the bone marrow.
Blood smear	Pseudothrombocytopenia, schistocytes, dysplasia, blasts, general cell line; changes and maturation
Substrates (folate, Vitamin B12/holoTC, iron tests)	Substrate deficiency
PT, INR, aPTT, Fibrinogen	Coagulopathy (DIC, TTP)
Liver and kidney tests function	Liver or kidney disease
Infections (HIV, HCV, HBV, CMV, EBV, Parvo B19)	Viral infection
Protein electrophoresis with immunofixation	Lymphoid neoplasms, plasma cell neoplasms
Free light chains	plasma cell neoplasms
LDH, bilirubin, haptoglobin, DAT	Hemolysis
TSH (ANA, ANCA, RF)	Autoimmunity
Abdominal ultrasound	Liver disease, splenomegaly, lymph nodes enlargement

CBC: complete blood count; DAT: direct antiglobulin test; DIC: disseminated intravascular coagulation, holoTC: holotranscobalamin; IPF: immature platelet fraction; LDH: Lactate dehydrogenase; MPV: mean platelet volume; TTP: thrombotic thrombocytopenic purpura.

**Table 2:** Common findings and differences between hypoplastic myelodysplastic syndrome (hMDS) and aplastic anemia (AA) [52].

Parameters	Hypoplastic MDS	Aplastic Anemia
**Cytopenia**	Present	Present
**BM cellularity**	Hypocellular	Aplastic (<10% cellularity or significantly hypocellular)
**BM hematopoiesis**		
*Erythropoiesis*	Present	Present in nests, “hot spots”
*Granulopoiesis*	Present	Typically decreased
*Megakaryopoiesis*	Present	Decreased or absent
*BM fat replacement*	Possible	Typical
**Dysplasia**		
*Erythroid dysplasia*	Frequent	Possible
*Granulocytic dysplasia*	Frequent	Normal morphology
*Megakaryocytic dysplasia*	Frequent	Normal morphology
**Ring sideroblasts**	Possible	Absent
**Blasts**	Variable	Absent
CD34+ or CD117+ immunohistochemistry	Normal or increased	No increase
**Marrow fibrosis**	Possible	Absent
**PNH clone**	Unusual	Frequent
**Splenomegaly at diagnosis**	Possible	Absent
**Karyotype**	Abnormal ~50%	Clonal abnormality possible (~12%)
*Recurrent cytogenetic abnormalities*	-Y, del(11q), −5/del(5q), del(12p), del(20q), −7/del(7q), +8, +19, i(17q), inv(3)/t(3q)/del(3q)	At Diagnosis: del(13q), +8 Evolution: −7, −5/del (5q), del(20q)
*Complex cytogenetics* *(≥3 abnormalities)*	Possible	Absent
*Acquired CN-LOH*	Possible	Possible (<20%)
**Somatic mutated genes**	*SF3B1*, *SRSF2*, *U2AF1*, *ZRSR2*, *TET2*, *DNMT3A*, *IDH1*, *IDH2*, *ASXL1*, *EZH2*, *RUNX1*, *NRAS*, *BCOR*, *TP53*, *STAG2*	Particularly *PIGA*, *ASXL1*, *BCOR*, *BCORL1*; 5–52% of patients will present MDS-associated mutations
**Germline mutations**	Should be investigated in patients with suspicion of underlying germline predisposition.	Should be investigated in patients with suspicion of underlying congenital BMF.

BM: bone marrow; PNH: paroxysmal nocturnal hemoglobinuria CN-LOH: copy number-neutral loos of heterozygosity.

**Table 3:** Integrated cyto-histologic/genetic score (hg-score) for distinction of hypoplastic myelodysplastic syndrome (hMDS) and aplastic anemia (AA) [26].

Cytological/Histological Variables	
**Requisite criteria**	**Scoring points**
Bone marrow blasts AND/OR CD34 + cells ≥5%	2
Bone marrow blasts AND/OR CD34 + cells 2–4%	1
Fibrosis grade 2–3	1
Dysmegakaryopoiesis	1
**Co-criteria**	
Ring sideroblasts ≥15%	2
Ring sideroblasts 5–14% *	1
Severe dysgranulopoiesis	1
**Karyotype (co-criterion)**	
Presumptive cytogenetic abnormality *	2
**Somatic mutation (co-criterion)**	
Specific high-risk mutation pattern **	1

* According to World Health Organization (WHO) criteria [53] ** According to Malcovati et al. [79] Receiver Operating Characteristic (ROC) analysis confirmed that a cutoff hg-score of 2 is associated with the highest percentage of correctly classified (Area Under the Curve, (AUC) 0.89, *p* < 0.001).

**Table 4:** Minimal diagnostic criteria for myelodysplastic syndromes (MDS) [80].

Criteria	Major Diagnostic Tests
**Prerequisite criteria (both must be fulfilled)**	
Constant cytopenia	Blood counts (over 6 months)
Exclusion of all other diseases as primary cause of cytopenia⁄dysplasia	BM smear and BM histology, cytogenetics, flow cytometry, molecular markers, other relevant investigations *
**MDS-related criteria (one of these must be fulfilled)**	
Morphological dysplasia in one of the three major lineages	BM and PB smear, in certain situations BM histology
Blast count ≥5%	BM smear and histology
Ring sideroblasts ≥15% or ≥5% and SF3B1 mutation	Iron staining
Typical karyotype anomaly	Conventional karyotyping and/or FISH
**Co-criteria**	
Monoclonality of myeloid cells	Molecular markers and mutations
BM stem cell function	Circulating CFC, reticulocytes
Abnormal immunophenotype of BM cells	Multicolor flow cytometry, immunohistochemistry
Abnormal gene expression profile	mRNA profiling assays

BM, bone marrow; PB, peripheral blood; FISH, fluorescence in situ hybridization; CFC, colony-forming progenitor cells. * Investigations depend on the case history and overall situation in each case, and should always include a complete chemistry profile with inflammation parameters, immunoglobulins, a serum erythropoietin level, and a serum tryptase level.

**Table 5:** WHO 2016 classification for Myelodysplastic Syndromes (MDS) [53].

Subtype ^1^	No. of Dysplastic Lineages	No. of Cytopenic Lineages ^2^	% RS of all Erythroid Cells in BM		% Blasts in PB or BM AR: Auer Rods			Conventional Cytogenetics
			**wt*SF3B1***	**m*SF3B1***	**BM**	**PB**	**AR**	
**MDS-SLD**	1	1 or 2	<15	<5	<5	<1	-	
**MDS-MLD**	2 or 3	1–3	<15	<5	<5	<1	-	
**MDS RS-SLD**	1	1 or 2	≥15	≥5	<5	<1	-	
**MDS RS-MLD**	2 or 3	1–3	≥15	≥5	<5	<1	-	
**MDS del(5q)**	1–3	1 or 2	n.a.	n.a.	<5	<1	-	Isolated del(5q) +/− 1 add. aberration without del(7q)/−7
**MDS EB-1**	0–3	1–3	n.a.	n.a.	5–9	2–4	-	
**MDS EB-2**	0–3	1–3	n.a.	n.a.	10–19	5–19	+	
**MDS-U**			<15	<5	<5	<1	-	
**(a) 1% blasts in PB**	1–3	1–3	n.a.	n.a.	<5	1 ^3^	-	
**(b) SLD with pancytopenia**	1	3	n.a.	n.a.	<5	<1	-	
**(c) defining cytogenetic aberration**	0	1–3	<15 ^4^	n.a.	<5	<1	-	MDS defining cytogenetic aberration
**RCC**	1–3	1–3	<15	≤5	<5	<1	-	

SLD: single-lineage dysplasia; MLD: multilineage dysplasia; RS: ring sideroblasts; EB: excess of blasts; RCC: refractory cytopenia of the childhood; wt/*mSF3B1*: wild type or mutated *SF3B1*; PB: peripheral blood; BM: bone marrow; AR: Auer rods. ^1^ Without previous cytotoxic treatment or germline predisposition for myeloid neoplasms. ^2^ Cytopenias: hemoglobin <100 g/L, thrombocytes <100 G/L, neutrophils <1.8 G/L, monocytes <1 G/L. ^3^ 1% blasts in PB must be confirmed with a 2nd analysis. ^4^ ≥15% RS corresponds to MDS-RS-SLD. CAVE: If ≥50% are erythroid precursors and ≥20% blast cells of non-erythroid-lineage but <20% of all cells, this corresponds now to MDS (MDS-SLD/MLD or EB) and not any more to AML M6 erythroid/myeloid.

## Data Availability

Not applicable.

## Supplementary Materials

The following are available online at https://www.mdpi.com/2077-0383/10/5/1026/s1, Table S1: Overview of some of the typical germline mutations in different bone marrow failure entities.

## References

[1] Neunert C., Terrell D.R., Arnold D.M., Buchanan G., Cines D.B., Cooper N., Cuker A., Despotovic J.M., George J.N., Grace R.F. (2019). American Society of Hematology 2019 guidelines for immune thrombocytopenia. Blood Adv..

[2] Roman E., Smith A., Appleton S., Crouch S., Kelly R., Kinsey S., Cargo C., Patmore R. (2016). Myeloid malignancies in the real-world: Occurrence, progression and survival in the UK’s population-based Haematological Malignancy Research Network 2004–15. Cancer Epidemiol..

[3] Hellström-Lindberg E., Tobiasson M., Greenberg P. (2020). Myelodysplastic syndromes: Moving towards personalized management. Haematologica.

[4] Corey S.J., Minden M.D., Barber D.L., Kantarjian H., Wang J.C.Y., Schimmer A.D. (2007). Myelodysplastic syndromes: The complexity of stem-cell diseases. Nat. Rev. Cancer.

[5] Steensma D.P. (2018). Clinical Implications of Clonal Hematopoiesis. Mayo Clin. Proc..

[6] Steensma D.P. (2017). Predicting therapy-related myeloid neoplasms-and preventing them?. Lancet Oncol..

[7] Neukirchen J., Schoonen W.M., Strupp C., Gattermann N., Aul C., Haas R., Germing U. (2011). Incidence and prevalence of myelodysplastic syndromes: Data from the Düsseldorf MDS-registry. Leuk. Res..

[8] Foran J.M., Shammo J.M. (2012). Clinical Presentation, Diagnosis, and Prognosis of Myelodysplastic Syndromes. Am. J. Med..

[9] Strom S.S., Gu Y., Gruschkus S.K., Pierce S., Estey E.H. (2005). Risk factors of myelodysplastic syndromes: A case–control study. Leukemia.

[10] Bonadies N., Feller A., Rovo A., Ruefer A., Blum S., Gerber B., Stuessi G., Benz R., Cantoni N., Holbro A. (2017). Trends of classification, incidence, mortality, and survival of MDS patients in Switzerland between 2001 and 2012. Cancer Epidemiol..

[11] Rollison D.E., Howlader N., Smith M.T., Strom S.S., Merritt W.D., Ries L.A., Edwards B.K., List A.F. (2008). Epidemiology of myelodysplastic syndromes and chronic myeloproliferative disorders in the United States, 2001–2004, using data from the NAACCR and SEER programs. Blood.

[12] Babushok D.V., Bessler M. (2015). Genetic predisposition syndromes: When should they be considered in the work-up of MDS?. Best Pract. Res. Clin. Haematol..

[13] Steensma D.P., Bennett J.M. (2006). The Myelodysplastic Syndromes: Diagnosis and Treatment. Mayo Clin. Proc..

[14] Thota S., Gerds A.T. (2017). Myelodysplastic and myeloproliferative neoplasms: Updates on the overlap syndromes. Leuk. Lymphoma.

[15] Kipfer B., Daikeler T., Kuchen S., Hallal M., Andina N., Allam R., Bonadies N. (2018). Increased cardiovascular comorbidities in patients with myelodysplastic syndromes and chronic myelomonocytic leukemia presenting with systemic inflammatory and autoimmune manifestations. Semin. Hematol..

[16] Mekinian A., Grignano E., Braun T., Decaux O., Liozon E., Costedoat-Chalumeau N., Kahn J.-E., Hamidou M., Park S., Puéchal X. (2016). Systemic inflammatory and autoimmune manifestations associated with myelodysplastic syndromes and chronic myelomonocytic leukaemia: A French multicentre retrospective study. Rheumatology.

[17] Risitano A.M., Maciejewski J.P., Green S., Plasilova M., Zeng W., Young N.S. (2004). In-vivo dominant immune responses in aplastic anaemia: Molecular tracking of putatively pathogenetic T-cell clones by TCR beta-CDR3 sequencing. Lancet.

[18] Young N.S. (2013). Current concepts in the pathophysiology and treatment of aplastic anemia. Hematol. Am. Soc. Hematol. Educ. Program.

[19] Min K.-W., Jung H.Y., Han H.S., Hwang T.S., Kim S.-Y., Kim W.S., Lim S.D., Kim W.Y. (2014). Ileal mass-like lesion induced by Epstein-Barr virus-associated hemophagocytic lymphohistiocytosis in a patient with aplastic anemia. APMIS.

[20] Brown K.E., Idrees M., Shah S.A., Butt S., Butt A.M., Ali L., Hussain A., Rehman I.U., Ali M. (1997). Hepatitis-associated aplastic anemia. N. Engl. J. Med..

[21] Young N.S. (1990). Flaviviruses and bone marrow failure. JAMA.

[22] Locasciulli A., Bacigalupo A., Bruno B., Montante B., Marsh J., Tichelli A., Socié G., Passweg J. (2010). Hepatitis-associated aplastic anaemia: Epidemiology and treatment results obtained in Europe. A report of The EBMT aplastic anaemia working party. Br. J. Haematol..

[23] Calado R.T., Cooper J.N., Padilla-Nash H.M., Sloand E.M., Wu C.O., Scheinberg P., Ried T., Young N.S. (2011). Short telomeres result in chromosomal instability in hematopoietic cells and precede malignant evolution in human aplastic anemia. Leukemmia.

[24] Dumitriu B., Feng X., Townsley D.M., Ueda Y., Yoshizato T., Calado R.T., Yang Y., Wakabayashi Y., Kajigaya S., Ogawa S. (2015). Telomere attrition and candidate gene mutations preceding monosomy 7 in aplastic anemia. Blood.

[25] Townsley D.M., Dumitriu B., Young N.S. (2014). Bone marrow failure and the telomeropathies. Blood.

[26] Bono E., McLornan D., Travaglino E., Gandhi S., Gallì A., Khan A.A., Kulasekararaj A.G., Boveri E., Raj K., Elena C. (2019). Clinical, histopathological and molecular characterization of hypoplastic myelodysplastic syndrome. Leukemia.

[27] Stanley N., Olson T.S., Babushok D.V. (2017). Recent advances in understanding clonal haematopoiesis in aplastic anaemia. Br. J. Haematol..

[28] Kulasekararaj A.G., Jiang J., Smith A.E., Mohamedali A.M., Mian S., Gandhi S., Gaken J., Czepulkowski B., Marsh J.C., Mufti G.J. (2014). Somatic mutations identify a subgroup of aplastic anemia patients who progress to myelodysplastic syndrome. Blood.

[29] Triemstra J., Pham A., Rhodes L., Waggoner D.J., Onel K. (2015). A Review of Fanconi Anemia for the Practicing Pediatrician. Pediatr. Ann..

[30] Farooqui S.M., Ward R., Aziz M. (2020). Shwachman-Diamond Syndrome. StatPearls.

[31] Geddis A.E. (2006). Inherited Thrombocytopenia: Congenital Amegakaryocytic Thrombocytopenia and Thrombocytopenia with Absent Radii. Semin. Hematol..

[32] Kojima S. (2017). Why is the incidence of aplastic anemia higher in Asia?. Expert Rev. Hematol..

[33] Young N.S., Kaufman D.W. (2008). The epidemiology of acquired aplastic anemia. Haematologica.

[34] Song E.Y., Kang H.J., Shin H.Y., Ahn H.S., Kim I., Yoon S.-S., Park S., Kim B.K., Park M.H. (2010). Association of human leukocyte antigen class II alleles with response to immunosuppressive therapy in Korean aplastic anemia patients. Hum. Immunol..

[35] Sugimori C., Yamazaki H., Feng X., Mochizuki K., Kondo Y., Takami A., Chuhjo T., Kimura A., Teramura M., Mizoguchi H. (2007). Roles of DRB1 *1501 and DRB1 *1502 in the pathogenesis of aplastic anemia. Exp. Hematol..

[36] Yoshida N., Yagasaki H., Takahashi Y., Yamamoto T., Liang J., Wang Y., Tanaka M., Hama A., Nishio N., Kobayashi R. (2008). Clinical impact of HLA-DR15, a minor population of paroxysmal nocturnal haemoglobinuria-type cells, and an aplastic anaemia-associated autoantibody in children with acquired aplastic anaemia. Br. J. Haematol..

[37] Wang M., Nie N., Feng S., Shi J., Ge M., Li X., Shao Y., Huang J., Zheng Y. (2014). The polymorphisms of human leukocyte antigen loci may contribute to the susceptibility and severity of severe aplastic anemia in Chinese patients. Hum. Immunol..

[38] Jeong T.-D., Mun Y.-C., Chung H.-S., Seo D., Im J., Huh J. (2016). Novel deletion mutation of HLA-B*40:02 gene in acquired aplastic anemia. HLA.

[39] Maluf E., Hamerschlak N., Cavalcanti A.B., Júnior Á.A., Eluf-Neto J., Falcão R.P., Lorand-Metze I.G., Goldenberg D., Santana C.L., Rodrigues D.D.O.W. (2009). Incidence and risk factors of aplastic anemia in Latin American countries: The LATIN case-control study. Haematology.

[40] Yoshizato T., Dumitriu B., Hosokawa K., Makishima H., Yoshida K., Townsley D., Sato-Otsubo A., Sato Y., Liu D., Suzuki H. (2015). Somatic Mutations and Clonal Hematopoiesis in Aplastic Anemia. N. Engl. J. Med..

[41] Kallen M.E., Dulau-Florea A., Wang W., Calvo K.R. (2019). Acquired and germline predisposition to bone marrow failure: Diagnostic features and clinical implications. Semin. Hematol..

[42] Killick S.B., Bown N., Cavenagh J., Dokal I., Foukaneli T., Hill A., Hillmen P., Ireland R., Kulasekararaj A.G., Mufti G.J. (2016). Guidelines for the diagnosis and management of adult aplastic anaemia. Br. J. Haematol..

[43] Shimamura A., Alter B.P. (2010). Pathophysiology and management of inherited bone marrow failure syndromes. Blood Rev..

[44] Invernizzi R., Quaglia F., Della Porta M.G. (2015). Importance of classical morphology in the diagnosis of myelodysplastic syndrome. Mediterr. J. Hematol. Infect. Dis..

[45] Greenberg P., Cox C., Lebeau M.M., Fenaux P., Morel P., Sanz G., Sanz M., Vallespi T., Hamblin T., Oscier D. (1997). International scoring system for evaluating prognosis in myelodysplastic syndromes. Blood.

[46] Greenberg P.L., Tuechler H., Schanz J., Sanz G.F., Garcia-Manero G., Solé F., Bennett J.M., Bowen D., Fenaux P., Dreyfus F. (2012). Revised International Prognostic Scoring System for Myelodysplastic Syndromes. Blood.

[47] Bonadies N., Bacher V.U. (2019). What role can next-generation sequencing play in myelodysplastic syndrome care?. Expert Rev. Hematol..

[48] Silzle T., Blum S., Schuler E., Kaivers J., Rudelius M., Hildebrandt B., Gattermann N., Haas R., Germing U. (2019). Lymphopenia at diagnosis is highly prevalent in myelodysplastic syndromes and has an independent negative prog-nostic value in IPSS-R-low-risk patients. Blood Cancer J..

[49] Go R.S., Lust J.A., Phyliky R.L. (2003). Aplastic anemia and pure red cell aplasia associated with large granular lymphocyte leukemia. Semin. Hematol..

[50] Wang L., Zhou Y., Tang J., Zhan Q., Liao Y. (2019). CD4(−)/CD8(−)/CD56(+)/TCRgammadelta(+) T-cell large granular lymphocyte leukemia presenting as aplastic ane-mia: A case report and literature review. Zhonghua Xue Ye Xue Za Zhi.

[51] Tichelli A., Gratwohl A., Nissen C., Signer E., Gysi C.S., Speck B. (1992). Morphology in patients with severe aplastic anemia treated with antilymphocyte globulin. Blood.

[52] Rovó A., Ebmt O.B.O.T.S.-W., Tichelli A., Dufour C. (2012). Diagnosis of acquired aplastic anemia. Bone Marrow Transplant..

[53] Arber D.A., Orazi A., Hasserjian R., Thiele J., Borowitz M.J., Le Beau M.M., Bloomfield C.D., Cazzola M., Vardiman J.W. (2016). The 2016 revision to the World Health Organization classification of myeloid neoplasms and acute leukemia. Blood.

[54] Bain B.J., Clark D.M., Wilkins B.S. (2009). Bone Marrow Pathology.

[55] Bain B.J. (2001). Bone marrow trephine biopsy. J. Clin. Pathol..

[56] Rovo A., Kulasekararaj A., Medinger M., Chevallier P., Ribera J.M., Peffault de Latour R., Knol C., Iacobelli S., Kanfer E., Bruno B. (2019). Association of aplastic anaemia and lymphoma: A report from the severe aplastic anaemia working party of the Euro-pean Society of Blood and Bone Marrow Transplantation. Br. J. Haematol..

[57] Medinger M., Buser A., Stern M., Heim D., Halter J., Rovó A., Tzankov A., Tichelli A., Passweg J. (2012). Aplastic anemia in association with a lymphoproliferative neoplasm: Coincidence or causality?. Leuk. Res..

[58] Zonder J.A., Keating M., Schiffer C.A. (2002). Chronic lymphocytic leukemia presenting in association with aplastic anemia. Am. J. Hematol..

[59] Westers T.M., Ireland R., Kern W., Alhan C.C., Balleisen J.S., Bettelheim P., Burbury K., Cullen M., Cutler J., Della Porta M.G. (2012). Standardization of flow cytometry in myelodysplastic syndromes: A report from an international consortium and the European LeukemiaNet Working Group. Leukemia.

[60] Stojkov K., Silzle T., Stussi G., Schwappach D., Bernhard J., Bowen D., Čermák J., Dinmohamed A.G., Eeltink C., Eggmann S. (2020). Guideline-based indicators for adult patients with myelodysplastic syndromes. Blood Adv..

[61] Brodsky R.A. (2014). Paroxysmal nocturnal hemoglobinuria. Blood.

[62] Schanz J., Tüchler H., Solé F., Mallo M., Luño E., Cervera J., Granada I., Hildebrandt B., Slovak M.L., Ohyashiki K. (2012). New Comprehensive Cytogenetic Scoring System for Primary Myelodysplastic Syndromes (MDS) and Oligoblastic Acute Myeloid Leukemia After MDS Derived from an International Database Merge. J. Clin. Oncol..

[63] Gupta V., Brooker C., Tooze J.A., Yi Q.-L., Sage D., Turner D., Kangasabapathy P., Marsh J.C.W. (2006). Clinical relevance of cytogenetic abnormalities at diagnosis of acquired aplastic anaemia in adults. Br. J. Haematol..

[64] Hosokawa K., Katagiri T., Sugimori N., Ishiyama K., Sasaki Y., Seiki Y., Sato-Otsubo A., Sanada M., Ogawa S., Nakao S. (2012). Favorable outcome of patients who have 13q deletion: A suggestion for revision of the WHO ’MDS-U’ designation. Haematologica.

[65] Holbro A., Jotterand M., Passweg J.R., Buser A., Tichelli A., Rovó A. (2013). Comment to “Favorable outcome of patients who have 13q deletion: A suggestion for revision of the WHO ’MDS-U’ designation”. Haematologica.

[66] Heinrichs S., Li C., Look A.T. (2010). SNP array analysis in hematologic malignancies: Avoiding false discoveries. Blood.

[67] Ouahchi I., Zhang L., Brito R.B., Benz R., Müller R., Bonadies N., Tchinda J. (2019). Microarray-based comparative genomic hybridisation reveals additional recurrent aberrations in adult patients evaluated for myelodysplastic syndrome with normal karyotype. Br. J. Haematol..

[68] Kamps R., Brandão R.D., Bosch B.J.V.D., Paulussen A.D.C., Xanthoulea S., Blok M.J., Romano A. (2017). Next-Generation Sequencing in Oncology: Genetic Diagnosis, Risk Prediction and Cancer Classification. Int. J. Mol. Sci..

[69] Braggio E., Egan J.B., Fonseca R., Stewart A.K. (2013). Lessons from next-generation sequencing analysis in hematological malignancies. Blood Cancer J..

[70] Merker J.D., Valouev A., Gotlib J. (2012). Next-generation sequencing in hematologic malignancies: What will be the dividends?. Ther. Adv. Hematol..

[71] Chirnomas S.D., Kupfer G.M. (2013). The Inherited Bone Marrow Failure Syndromes. Pediatr. Clin. N. Am..

[72] Mangaonkar A.A., Patnaik M.M. (2020). Hereditary Predisposition to Hematopoietic Neoplasms: When Bloodline Matters for Blood Cancers. Mayo Clin. Proc..

[73] Wegman-Ostrosky T., Savage S.A. (2017). The genomics of inherited bone marrow failure: From mechanism to the clinic. Br. J. Haematol..

[74] Alter B.P. (2007). Diagnosis, Genetics, and Management of Inherited Bone Marrow Failure Syndromes. Hematol. Am. Soc. Hematol. Educ. Program..

[75] Walne A.J., Collopy L., Cardoso S., Ellison A., Plagnol V., Albayrak C., Albayrak D., Kilic S.S., Patıroglu T., Akar H. (2016). Marked overlap of four genetic syndromes with dyskeratosis congenita confounds clinical diagnosis. Haematologica.

[76] Bluteau O., Sebert M., Leblanc T., De Latour R.P., Quentin S., Lainey E., Hernandez L., Dalle J.-H., De Fontbrune F.S., Lengline E. (2018). A landscape of germ line mutations in a cohort of inherited bone marrow failure patients. Blood.

[77] Ogawa S. (2019). Genetics of MDS. Blood.

[78] Li M.M., Chao E., Esplin E.D., Miller D.T., Nathanson K.L., Plon S.E., Scheuner M.T., Stewart D.R., ACMG Professional Practice and Guidelines Committee (2020). Points to consider for reporting of germline variation in patients undergoing tumor testing: A statement of the Ameri-can College of Medical Genetics and Genomics (ACMG). Genet. Med..

[79] Malcovati L., Gallì A., Travaglino E., Ambaglio I., Rizzo E., Molteni E., Elena C., Ferretti V.V., Catricalà S., Bono E. (2017). Clinical significance of somatic mutation in unexplained blood cytopenia. Blood.

[80] Valent P., Orazi A., Steensma D.P., Ebert B.L., Haase D., Malcovati L., Van De Loosdrecht A.A., Haferlach T., Westers T.M., Wells D.A. (2017). Proposed minimal diagnostic criteria for myelodysplastic syndromes (MDS) and potential pre-MDS conditions. Oncotarget.

[81] Malcovati L., Germing U., Kuendgen A., Della Porta M.G., Pascutto C., Invernizzi R., Giagounidis A., Hildebrandt B., Bernasconi P., Knipp S. (2007). Time-Dependent Prognostic Scoring System for Predicting Survival and Leukemic Evolution in Myelodysplastic Syndromes. J. Clin. Oncol..

[82] Charlson M.E., Pompei P., Ales K.L., MacKenzie C. (1987). A new method of classifying prognostic comorbidity in longitudinal studies: Development and validation. J. Chronic Dis..

[83] Sorror M.L., Maris M.B., Storb R., Baron F., Sandmaier B.M., Maloney D.G., Storer B. (2005). Hematopoietic cell transplantation (HCT)-specific comorbidity index: A new tool for risk assessment before alloge-neic HCT. Blood.

[84] Sorror M.L., Storb R.F., Sandmaier B.M., Maziarz R.T., Pulsipher M.A., Maris M.B., Bhatia S., Ostronoff F., Deeg H.J., Syrjala K.L. (2014). Comorbidity-Age Index: A Clinical Measure of Biologic Age Before Allogeneic Hematopoietic Cell Transplantation. J. Clin. Oncol..

[85] Della Porta M.G., Malcovati L., Strupp C., Ambaglio I., Kuendgen A., Zipperer E., Travaglino E., Invernizzi R., Pascutto C., Lazzarino M. (2011). Risk stratification based on both disease status and extra-hematologic comorbidities in patients with myelo-dysplastic syndrome. Haematologica.

[86] Swinkels M., Rijkers M., Voorberg J., Vidarsson G., Leebeek F.W.G., Jansen A.J.G. (2018). Emerging Concepts in Immune Thrombocytopenia. Front. Immunol..

[87] Waisbren J., Dinner S., Altman J., Frankfurt O., Helenowski I., Gao J., McMahon B.J., Stein B.L. (2016). Disease characteristics and prognosis of myelodysplastic syndrome presenting with isolated thrombocytopenia. Int. J. Hematol..

[88] Kuroda J., Kimura S., Kobayashi Y., Wada K., Uoshima N., Yoshikawa T. (2002). Unusual myelodysplastic syndrome with the initial presentation mimicking idiopathic thrombocytopenic purpura. Acta Haematol..

[89] Bryan J., Jabbour E., Prescott H., Kantarjian H. (2010). Thrombocytopenia in patients with myelodysplastic syndromes. Semin. Hematol..

[90] Mittelman M. (2018). Good news for patients with myelodysplastic syndromes and thrombocytopenia. Lancet Haematol..

[91] Manoharan A., Brighton T., Gemmell R., Lopez K., Moran S., Kyle P. (2002). Platelet Dysfunction in Myelodysplastic Syndromes: A Clinicopathological Study. Int. J. Hematol..

[92] Galera P., Dulau-Florea A., Calvo K.R. (2019). Inherited thrombocytopenia and platelet disorders with germline predisposition to myeloid neoplasia. Int. J. Lab. Hematol..

[93] Sekeres M.A., Schoonen W.M., Kantarjian H., List A., Fryzek J., Paquette R., Maciejewski J.P. (2008). Characteristics of US Patients with Myelodysplastic Syndromes: Results of Six Cross-sectional Physician Surveys. J. Natl. Cancer Inst..

[94] Klymenko S.V., Belyi D.A., Ross J.R., Owzar K., Jiang C., Li Z., Minchenko J.N., Kovalenko A.N., Bebeshko V.G., Chao N.J. (2011). Hematopoietic cell infusion for the treatment of nuclear disaster victims: New data from the Chernobyl accident. Int. J. Radiat. Biol..

[95] Camitta B.M., Rappeport J.M., Parkman R., Nathan D.G. (1975). Selection of patients for bone marrow transplantation in severe aplastic anemia. Blood.

[96] Bacigalupo A., Hows J., Gluckman E., Nissen C., Marsh J., Van Lint M.T., Congiu M., De Planque M.M., Ernst P., McCann S. (1988). Bone marrow transplantation (BMT) versus immunosuppression for the treatment of severe aplastic anaemia (SAA): A report of the EBMT SAA Working Party. Br. J. Haematol..

[97] Gross S., Kiwanuka J. (1981). Chronic ITP terminating in aplastic anemia. Am. J. Pediatr. Hematol. Oncol..

[98] Yun G.-W., Yang Y.-J., Song I.-C., Baek S.-W., Lee K.-S., Lee H.-J., Yun H.-J., Kwon K.-C., Kim S., Jo D.-Y. (2011). Long-term outcome of isolated thrombocytopenia accompanied by hypocellular marrow. Korean J. Hematol..

[99] Levy I., Laor R., Jiries N., Bejar J., Polliack A., Tadmor T. (2018). Amegakaryocytic Thrombocytopenia and Subsequent Aplastic Anemia Associated with Apparent Epstein-Barr Virus Infection. Acta Haematol..

[100] King J.A.C., ElKhalifa M.Y., Latour L.F. (1997). Rapid Progression of Acquired Amegakaryocytic Thrombocytopenia to Aplastic Anemia. South. Med. J..

[101] Young N.S., Maciejewski J.P., Sloand E., Chen G., Zeng W., Risitano A., Miyazato A. (2002). The relationship of aplastic anemia and PNH. Int. J. Hematol..

[102] Pu J.J., Mukhina G., Wang H., Savage W.J., Brodsky R.A. (2011). Natural history of paroxysmal nocturnal hemoglobinuria clones in patients presenting as aplastic anemia. Eur. J. Haematol..

[103] Sutherland D.R., Illingworth A., Marinov I., Ortiz F., Andrea I., Payne D., Wallace P.K., Keeney M. (2018). ICCS/ESCCA Consensus Guidelines to detect GPI-deficient cells in Paroxysmal Nocturnal Hemoglobinuria (PNH) and related Disorders Part 2—Reagent Selection and Assay Optimization for High-Sensitivity Testing. Cytom. Part B Clin. Cytom..

[104] Auerbach A.D. (2009). Fanconi anemia and its diagnosis. Mutat. Res. Mol. Mech. Mutagen..

[105] Malcovati L., Hellström-Lindberg E., Bowen D., Adès L., Cermak J., Del Cañizo C., Della Porta M.G., Fenaux P., Gattermann N., Germing U. (2013). Diagnosis and treatment of primary myelodysplastic syndromes in adults: Recommendations from the European LeukemiaNet. Blood.

[106] Abdel-Wahab O., Figueroa M.E. (2012). Interpreting new molecular genetics in myelodysplastic syndromes. Haematology.

[107] Heuser M., Thol F., Ganser A. (2016). Clonal Hematopoiesis of Indeterminate Potential. Dtsch. Aerzteblatt Int..

[108] Jaiswal S., Fontanillas P., Flannick J., Manning A., Grauman P.V., Mar B.G., Lindsley R.C., Mermel C.H., Burtt N., Chavez A. (2014). Age-Related Clonal Hematopoiesis Associated with Adverse Outcomes. N. Engl. J. Med..

[109] Genovese G., Kähler A.K., Handsaker R.E., Lindberg J., Rose S.A., Bakhoum S.F., Chambert K., Mick E., Neale B.M., Fromer M. (2014). Clonal Hematopoiesis and Blood-Cancer Risk Inferred from Blood DNA Sequence. N. Engl. J. Med..

[110] Xie M., Lu C., Wang J., McLellan M.D., Johnson K.J., Wendl M.C., McMichael J.F., Schmidt H.K., Yellapantula V., Miller C.A. (2014). Age-related mutations associated with clonal hematopoietic expansion and malignancies. Nat. Med..

[111] Steensma D.P., Bejar R., Jaiswal S., Lindsley R.C., Sekeres M.A., Hasserjian R.P., Ebert B.L. (2015). Clonal hematopoiesis of indeterminate potential and its distinction from myelodysplastic syndromes. Blood.

