# Supplementary material for: When Should We Think of Myelodysplasia or Bone Marrow Failure in a Thrombocytopenic Patient? A Practical Approach to Diagnosis
Source: J Clin Med. 2021 Mar 2;10(5):1026. doi: 10.3390/jcm10051026 (PMC7958851; doi:10.3390/jcm10051026)
Supplement: Supplementary file 1 [file jcm-10-01026-s001.pdf]

Special Issue "Latest Clinical Advances of Thrombocytopenia"; Journal of Clinical Medicine

When Should We Think of Myelodysplasia or Bone Marrow Failure in a Thrombocytopenic Patient? A Practical Approach to Diagnosis

Nicolas Bonadies [1,2,†], Alicia Rovó [1,†], Naomi Porret [1] and Ulrike Bacher [1,*]

Online Supplement

Supplemental Table S1: Overview of some of the typical germline mutations in different bone marrow failure entities.

Disease entity	Affected genes
**Fanconi anemia (FA)**	*BRCA1, BRCA2, BRIP1, ERCC4, FANCA, FANCB, FANCC, FANCD2, FANCE, FANCF, FANCG, FANCI, FANCL, FANCM, MAD2L2, PALB2, RAD51, RAD51C, RFWD3, SLX4, UBE2T, XRCC2*
**Hereditary anemia**	*AMN, ATRX, CDAN1, CUBN, G6PD, GPI, KCNA4, PGK1, PKLR, SBDS, SEC23B, SLC11A2, TCN2, TMPRSS6, TPI1*
**Diamond Blackfan anemia (DBA)**	*ADA2, GATA1, RPL11, RPL26, RPL35A, RPL5, RPS7, RPS10, RPS17, RPS19, RPS24, RPS26*
**Telomere syndromes**	*ACD, CTC1, DKC1, NAF1, NHP2, NOP10, PARN, RTEL1, SRP72, TERC, TERT, TINF2, USB1, WRAP53*
**Hereditary neutropenia**	*AK2, CSF3R, ELANE/ELA2, G6PC3, GATA2, GFI1, HAX1, LAMTOR2/MAPBPIP (p14), SAMD9, SAMD9L, SRP72, WAS*
**Hereditary thrombocytopenia/thrombo-cytopathia**	*ACTN1, ANKRD26, ANO6, AP3B1, BLOC1S3, CYCS, DTNBP1, ETV6, FERMT3, FLI1, FLNA, GATA1, GFI1B, GGCX, GNE, GP1BA, GP1BB, GP6, GP9, HOXA11, HPS1, HPS3, HPS4, HPS5, HPS6, ITGA2, ITGA2B, ITGB3, LYST, MPL, MYH9, NBEA, NBEAL2, ORAI1, P2RY12, PLA2G4A, PLAU, RASGRP2, RBM8A, RUNX1, STIM1, STXBP2, TBXA2R, TBXAS1, THPO, VIPAS39, VPS33B*
**Hereditary myeloid neoplasia**	*ANKRD26* (promotor region), *CEBPA, DDX41, ETV6, GATA2, KRAS, PRF1, RUNX1, TP53*
